# Sporoderm-removed Ganoderma lucidum spores ameliorated early depression-like behavior in a rat model of sporadic Alzheimer’s disease

**DOI:** 10.3389/fphar.2024.1406127

**Published:** 2024-04-24

**Authors:** Yan Zhao, Yu Qin, Xiao Hu, Xi Chen, Yan-Ping Jiang, Xue-Jun Jin, Gao Li, Zhen-Hao Li, Ji-Hong Yang, Su-Ying Cui, Yong-He Zhang

**Affiliations:** ^1^ Key Laboratory of Natural Medicines of the Changbai Mountain, Ministry of Education, College of Pharmacy, Yanbian University, Yanji, China; ^2^ Department of Pharmacy, Yanbian University Hospital, Yanji, China; ^3^ Department of Pharmacology, School of Basic Medical Science, Peking University, Beijing, China; ^4^ Zhejiang ShouXianGu Pharmaceutical Co. Ltd., Wuyi, China

**Keywords:** sporadic Alzheimer’s disease, depression, G. lucidum spores, microglia, NF-κB/NLRP3 pathway

## Abstract

**Introduction:** *Ganoderma lucidum*: (G. lucidum, Lingzhi) is a medicinal and edible homologous traditional Chinese medicine that is used to treat various diseases, including Alzheimer’s disease and mood disorders. We previously reported that the sporoderm-removed *G. lucidum* spore extract (RGLS) prevented learning and memory impairments in a rat model of sporadic Alzheimer’s disease (sAD), but the effect of RGLS on depression-like behaviors in this model and its underlying molecular mechanisms of action remain unclear.

**Method:** The present study investigated protective effects of RGLS against intracerebroventricular streptozotocin (ICV-STZ)-induced depression in a rat model of sAD and its underlying mechanism. Effects of RGLS on depression- and anxiety-like behaviors in ICV-STZ rats were assessed in the forced swim test, sucrose preference test, novelty-suppressed feeding test, and open field test.

**Results:** Behavioral tests demonstrated that RGLS (360 and 720 mg/kg) significantly ameliorated ICV-STZ-induced depression- and anxiety-like behaviors. Immunofluorescence, Western blot and enzyme-linked immunosorbent assay results further demonstrated that ICV-STZ rats exhibited microglia activation and neuroinflammatory response in the medial prefrontal cortex (mPFC), and RGLS treatment reversed these changes, reflected by the normalization of morphological changes in microglia and the expression of NF-κB, NLRP3, ASC, caspase-1 and proinflammatory cytokines. Golgi staining revealed that treatment with RGLS increased the density of mushroom spines in neurons. This increase was associated with elevated expression of brain-derived neurotrophic protein in the mPFC.

**Discussion:** In a rat model of ICV-STZ-induced sAD, RGLS exhibits antidepressant-like effects, the mechanism of which may be related to suppression of the inflammatory response modulated by the NF-κB/NLRP3 pathway and enhancement of synaptic plasticity in the mPFC.

## 1 Introduction

Alzheimer’s disease (AD) is a common neurodegenerative disease in the elderly, characterized by progressive cognitive dysfunction and neuropsychiatric symptoms that affect nearly all patients (Querfurth and LaFerla, 2010). Among these neuropsychiatric symptoms, depression is the most common. More than 50% of AD patients present apparent depressive symptoms in the early course of the disease (Cassano et al., 2019). Depression has been recognized as a potential precursor symptom of AD (Cassano et al., 2019) and can increase the risk of behavioral disorders in AD patients, accelerate cognitive decline, reduce quality of life, cause disability, increase mortality, and increase direct economic costs (Milwain and Nagy, 2005; Burke et al., 2019). Therefore, treating and improving depression symptoms in AD patients can improve the quality of life of patients and possibly delay the course of the disease. However, conventional monoaminergic antidepressants have been shown to be ineffective in treating depressive symptoms in AD patients, and serious adverse effects have been observed (Orgeta et al., 2017). There is a need to discover more suitable therapeutic drugs to treat depression in AD patients. Some traditional herbal medicines in China have recently gained more attention, including *Ganoderma lucidum* (*G. lucidum*).

*G. lucidum* (named Lingzhi in China) has been widely used for thousands of years as a medicinal and edible homologous traditional medicine. It may calm the mind by nourishing one’s vitality and can be applied to treat restlessness, insomnia palpitation, cough and asthma, deficiency fatigue, shortness of breath, disinclination to eat (The State Pharmacopoeia Commission of People’s Republic of China, 2020). Ganoderma spore is the seed of *G. lucidum*. It has been found that Ganoderma spores contain similar bioactive molecules to those found in the fruit-body of *G. lucidum* and like its fruit body possess potential for medicinal application (Soccol et al., 2016). One challenge when extracting these constituents from spores is breaking the sturdy, thick wall, known as the sporoderm. With the development of sporoderm-breaking techniques, the medicinal value of *G. lucidum* spores has increased. Our previous study showed that breaking and removing the sporoderm of a *G. lucidum* spore extract (RGLS) significantly improved memory and cognitive impairments on day 14 in rats that were intracerebroventricularly injected with streptozotocin (ICV-STZ) (Zhao et al., 2021). ICV-STZ rats exhibited typical behavioral, neurochemical, and histological features that resemble sporadic AD (sAD) in humans (Grieb, 2016). In addition, ICV-STZ mice exhibited depression-like behavior on day 7 after ICV-STZ (Souza et al., 2017a), which was similar to clinical findings that depression precedes the onset of memory impairments in AD. These results suggest that ICV-STZ is a valid rodent model for studying early depression that is associated with sAD. However, the effects of RGLS on depression-like behavior have not been systematically investigated in a rodent model of sAD.

Patients with AD have extensive beta-amyloid (Aβ) deposition, Tau hyperphosphorylation, and the loss of neurons in brain regions that are associated with learning, memory, cognition, and emotion (Khan et al., 2020). Recent studies showed that the neuroinflammatory response plays an important role in the pathogenesis of sAD, especially in the early stage when animals exhibit depression-like behavior (Souza et al., 2017b). Depression may be related to significantly higher levels of inflammatory factors and microglia activation in the medial prefrontal cortex (mPFC) (Belleau et al., 2019). The upregulation of inflammatory factors and microglia activation were also observed in the PFC in ICV-STZ rats (Souza et al., 2022), but the underlying inflammatory response pathways in the mPFC in ICV-STZ-induced depression-like behavior remain unclear.

The nuclear factor κB (NF-κB)/NOD-like receptor containing pyrin-domain 3 (NLRP3) signaling pathway mediates the transduction of inflammation-related signals and the release of inflammatory factors through the recognition of exogenous pathogens, such as lipopolysaccharide, leading to neuronal damage and neurodegeneration (Li W. et al., 2021). This pathway has been extensively studied in animal models of AD and depression (Feng et al., 2020; Xia et al., 2023). Studies have shown that *G. lucidum* extracts improved depression-like behavior by exerting anti-inflammatory effects in social defeat (Li H. et al., 2021) and maternal separation models of depression (Mi et al., 2022) in rats. NLRP3-induced inflammation was suppressed by *G. lucidum* treatment in a Parkinson’s disease model in mice (Ren et al., 2022). These studies indicate that NLRP3-mediated inflammation might be a key component of the pharmacological mechanism of action of *G. lucidum*. The present study evaluated the pharmacological effects of RGLS on depression-like behavior in ICV-STZ rats and explored related mechanisms from the perspective of NF-κB/NLRP3-mediated neuroinflammation and neuroplasticity.

## 2 Materials and methods

### 2.1 Animals

Sprague-Dawley rats (200–220 g) were purchased from the Animal Center of Peking University Health Science Center (Beijing, China; certificate No. SCXK, 2006-0001). All animals were individually housed in plastic cages under a standard 12 h/12 h light/dark cycle (lights on at 9:00 p.m.) in a temperature-controlled room (temperature of 23°C ± 1 °C and humidity of 50% ± 10%). Food and water were available *ad libitum* unless otherwise specified. Animals were given 1 week to acclimatize to the new environment before surgery. All animals were handled daily. All experimental animal protocols were conducted in accordance with “Reporting animal research: Explanation and elaboration for the ARRIVE guidelines 2.0” (Percie du Sert et al., 2020) and approved by the Peking University Committee on Animal Care and Use (permission no. LA 2020279).

### 2.2 Surgery

For habituation, the animals were connected to the recording apparatus at least 1 day before the experiments. Rats were anesthetized with isoflurane (5% induction and 2% maintenance) and the heads were secured in a stereotaxic apparatus. The guide cannula (O.D. 0.64 mm × I.D. 0.25 mm, C.C. 1.2 mm, RWD Life Science Co. Ltd., Shenzhen, China) was directed toward the lateral cerebral ventricle at the following coordinates relative to the bregma from the stereotaxic atlas of Paxinos and Watson (Paxinos and Watson, 1986): AP = −0.8 mm, ML = −1.50 mm and DV = −3.50 mm. Dental acrylic cement and another three stainless steel screws were fixed to the skull in order to affix the cannula. A dummy stylet was inserted into the cannula to prevent obstruction. Subsequently, rats were kept warm until full recovery from anesthesia. Topical analgesic (lidocaine/prilocaine cream) and penicillin was used to prevent signs of pain and/or infection. All rats were handled once time daily after surgery. The rats were allowed to recover for at least 7 days before exposure to STZ.

### 2.3 Drug treatment and experimental design

STZ (Sigma-Aldrich, S0130, Saint Louis, Missouri, United States) was resolved in 4 μL artificial cerebrospinal fluid (aCSF; Tocris Bioscience, 3,525/25 mL, Bristol, UK) at a dose of 3 mg/kg prior to administration. STZ was slowly and continually injected in the ventricle using a Hamilton micro syringe attached to the injection cannula over 5 min. The needle was left in the same spot for an additional 3 min to allow for diffusion. STZ were microinjected at a rate of 0.8 μL/min.

RGLS was provided by Zhejiang ShouXianGu Pharmaceutical Co. Ltd. (batch No. 20181101, Zhejiang, China). As described in our previous report, this product was approved by the State Food and Drug Administration of China in 2016 (China health food approval No. G20160280) and the representative chromatograms of RGLS obtained by LC-QTOF-MS in negative ion mode were presented in our previous reports (Li et al., 2020; Zhao et al., 2021). The results showed that RGLS contained 99 triterpenes, 1 linoleic acid and 9 potential new compounds. The chemical composition and content of RGLS were much higher than that of *G. lucidum* spore powder extracts without broken wall. The voucher specimen (SXG-GLS-20181006016) was preserved in our laboratory and the company. The samples were kept in the Department of Pharmacology, School of Basic Medical Science, Peking University. RGLS was dissolved in distilled water to a final concentration of 45, 90 and 180 mg/mL. The dose of RGLS (180, 360, 720 mg/kg) used in this study.

**FIGURE 1:**
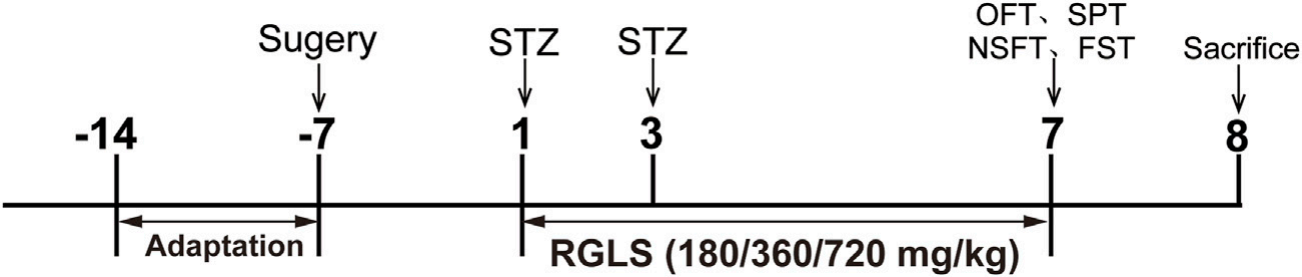
Experimental design.

The experimental design is presented in Figure 1. Rats were respectively assigned in a random manner to five groups: Vehicle; STZ; STZ + RGLS (180 mg/kg); STZ + RGLS (360 mg/kg) and STZ + RGLS (720 mg/kg). After 7 days of recovery from surgery, the STZ and STZ + RGLS groups received twice intracerebroventricularly administration of STZ at an interval of 48 h. The STZ + RGLS group was administered orally with 180 or 360 or 720 mg/kg RGLS by gavage daily from day 1 to day 7. Vehicle group was treated with aCSF intracerebroventricularly and distilled water intragastrically. Subsequently, all the behavioral tests were conducted on day 7. On day 8, the animals were decapitated, the mPFC region was rapidly dissected using a brain blade and frozen at −80 °C for subsequent biochemical determinations.

### 2.4 Depression- and anxiety-like behavior determination

All operations were conducted during the light phase, and each animal was used only once time in each behavioral test. Before the tests, animal was introduced to the experiment room to acclimate to the environment for at least 2 h. Depression-like behaviors of the animals were evaluated by the sucrose preference test, novelty-suppressed feeding test and forced swim test. Anxiety-like behaviors of the animals were evaluated by open field test.

#### 2.4.1 Open field test (OFT)

Spontaneous locomotion was measured by the OFT for a 10 min period beginning at 10:00 a.m. Briefly, animals were released into the central area of an open field (90 cm × 90 cm × 40 cm), and their activities were recorded by a video tracking system (DigBehv-LM4, Jiliang Software Technology, Shanghai, China) on top of the open field. The total distance was determined to measure the rat locomotor activities. And less time in the center is indicative of a more anxiety-like phenotype. The field was thoroughly cleaned with 75% alcohol after each recording session.

#### 2.4.2 Sucrose preference test (SPT)

The SPT was performed beginning at 11:00 a.m. Before test, rats were individually housed and habituated with two identical bottles filled with 1% sucrose solution for 48 h, then deprived food and water for 24 h. On day 7, rats were given a free choice between two bottles for 1 h, one filled with 1% sucrose solution and the other filled with water. Sucrose preference is a classical indicator of anhedonia-like behavior. For analyses, sucrose preference was calculated according to the following formula: Sucrose preference (%) = sucrose intake (g)/(sucrose intake [g] + water intake [g]) × 100%.

#### 2.4.3 Novelty-suppressed feeding test (NSFT)

The NSFT was performed beginning at 1:00 PM. Rats fasted 24 h before the test. NSFT was carried out in a circular field (122 cm diameter) and the test was begun by placing five to six pellets of food in the center of the arena. On day 7, a rat was placed in a quadrant of the arena and a video camera was used for observing and timing. The latency time for the rat from leaving the periphery of the arena to fetched the food using its forepaws and started eating was recorded. To exclude ingestion bias, home-cage food consumption was measured within 60 min of the test’s completion.

#### 2.4.4 Forced swim test (FST)

The procedure was based on our previous study. The FST was performed beginning at 3:00 PM. Briefly, animals were placed in a Plexiglas cylinder (60 cm height, 25 cm diameter) filled with tap water (24 ± 1 °C) to a depth of 40 cm for 5 min. A 15-min pretest was conducted at 10 a.m. on day 6. Immobility time was defined as floating or minimal movements of both limbs and tails that were required to keep the head above the water. Their performance was recorded by a video camera on top of the cylinder.

### 2.5 Western blot analysis

The rats were decapitated and mPFC tissue was homogenized in RIPA lysis buffer and complete protease and phosphatase inhibitors. Then the homogenate was followed by centrifugation at 12,000 g for 15 min at 4 °C. The 5 X loading buffer was added to the supernatant and then used to detect protein levels. The subsequent procedure was essentially the same to Zhao et al. (Zhao et al., 2021). Proteins of 40–50 μg isolated from tissues were resolved by 10% sodium dodecyl sulfate-polyacrylamide gel electrophoresis (SDS-PAGE), followed by transferring to polyvinylidene difluoride membranes (PVDF; Bio-Rad, United States). The membranes were blocked with 5% skim milk for 1 h at room temperature and then incubated at 4 °C overnight with primary antibodies against β-actin, (1:4,000, ABclonal, AC038, Wuhan, China), ionized calcium-binding adaptor molecule-1 (Iba1, 1:1,000, Cell Signaling Technology, 17,198, MA, United States), NF-κB (1:1,000, ABclonal, A2547, Wuhan, China), NLRP3 (1:1,000, Abcam, ab263899, Cambridge, UK), caspase-1 (1:1,000, Abcam, ab179515, Cambridge, UK), apoptosis-associated speck-like protein (ASC, 1:1,000, Abcam, ab180799, Cambridge, UK), and brain-derived growth factor (BDNF, 1:500, Abcam, ab108319, Cambridge, UK). After washing three times with Tris-buffered saline with Tween-20 (TBST), blots were incubated with horseradish peroxidase-conjugated secondary antibodies for 2 h at room temperature. Lastly, protein expression was detected with the ECL Enhanced Kit (ABclonal, RM00021, Wuhan, China), and the intensity of each blot was analyzed with ImageJ software.

### 2.6 Immunofluorescent staining

Rats were euthanized and transcardially perfused with 0.01 M phosphate buffered saline (PBS), followed by 4% paraformaldehyde (PFA) in PBS. The brains were harvested, post fixed in 4% PFA overnight at 4 °C, and then consecutively dehydrated with 20% and 30% sucrose solution in sequence until brain sank. Subsequently, the brains were cut to a thickness of 20 µm on a cryostat microtome (Leica Microsystems UK, Leica CM 1950, Milton Keynes, UK). The slides were washed with PBS twice for 5 min each and were then placed in metal bath at 96 °C containing 0.01 M citrate buffer (pH 6.0) for 5 min. After return to room temperature, slides were washed three times and permeabilized with 0.01 M PBS containing 0.1% Triton X-100 for 20 min. After blocking with 10% donkey serum for 40 min at room temperature, the slides were incubated with primary antibodies rabbit anti-Iba1 (1:500, Cell Signaling Technology, 17,198, MA, United States) at 4 °C overnight. Alexa Fluor 448-conjugated donkey anti-rabbit antibody (1:2000, Abcam, ab150073, Cambridge, UK) was used as the secondary antibody. Nuclei were stained with DAPI before slides were viewed using a Nikon DS-Ri2 microscope camera (Nikon, Tokyo, Japan). For quantitative analysis, Fiji software was used to measure integrated density. All images of each section were acquired at 200× magnification. The number of branches and end-points can reflect microglia complexity and distinguish the state of microglia. In brief, bidimensional images were converted to 8-bit images and adjusted for brightness-contrast before thresholding to minimize background noise. Following these steps, the images were processed with the despeckle function and skeletozine. Then, we run the analysis of the skeleton program.

### 2.7 Enzyme-linked immunosorbent assay (ELISA)

The generations of tumor necrosis factor-alpha (TNF-α), interleukin-6 (IL-6), and interleukin-1beta (IL-1β) in the mPFC were measured using ELISA kits (ABclonal, RK00029, RK00020, RK00009, Wuhan, China) according to manufacturer’s instructions. The optical density (OD) of each well was quantified at 450 nm with an ELISA plate reader (Thermo Fisher Scientific Multiskan Mk3, MA, United States). Results are shown as pg/mg of protein.

### 2.8 Golgi staining

Golgi-Cox staining was used to visualize dendrites and dendritic spines. Brain was stained using the FD Rapid Golgi Stain Kit (FD Neurotechnologies, PK401A, MD, United States). Brains were immersed in a mixture of Solution A (potassium dichromate and mercuric chloride) and Solution B (potassium chromate) in the dark for 2 weeks, and the tissue was then immersed in Solution C for 1 week, according to the manufacturer’s instructions. 150 μm slices were then mounted on gelatin-coated slides using additional Solution C, then left to dry in the dark overnight. After dehydration and decoloration, the slides were sealed with neutral balsam (Solarbio, G8590, China). Slides were stored in the dark. 3 dendrites per rat were traced, and a total of 5 rats per group were counted. The images were captured under Nikon Eclipse Ci-L microscope (Nikon, Tokyo, Japan) using DP controller software with 100×A/1.25 oil immersion lens. The density of dendritic spine and the dendritic spine morphologies in mPFC were analyzed using the ImageJ software.

### 2.9 Statistical analysis

Data analysis was performed by using GraphPad Prism (Version 8.0.1). The sample size was the number of independent values, and statistical analysis was performed using these independent values. The data from the western blot were normalized to control group values and determined as ‘Fold change’ in figures. Data were analyzed using one-way analysis of variance (ANOVA) with Tukey’s multiple comparison test. The results were considered significant at *p* < 0.05. All data were presented as the mean ± SEM.

## 3 Results

### 3.1 RGLS ameliorates ICV-STZ-induced depression- and anxiety-like behaviors

**FIGURE 2:**
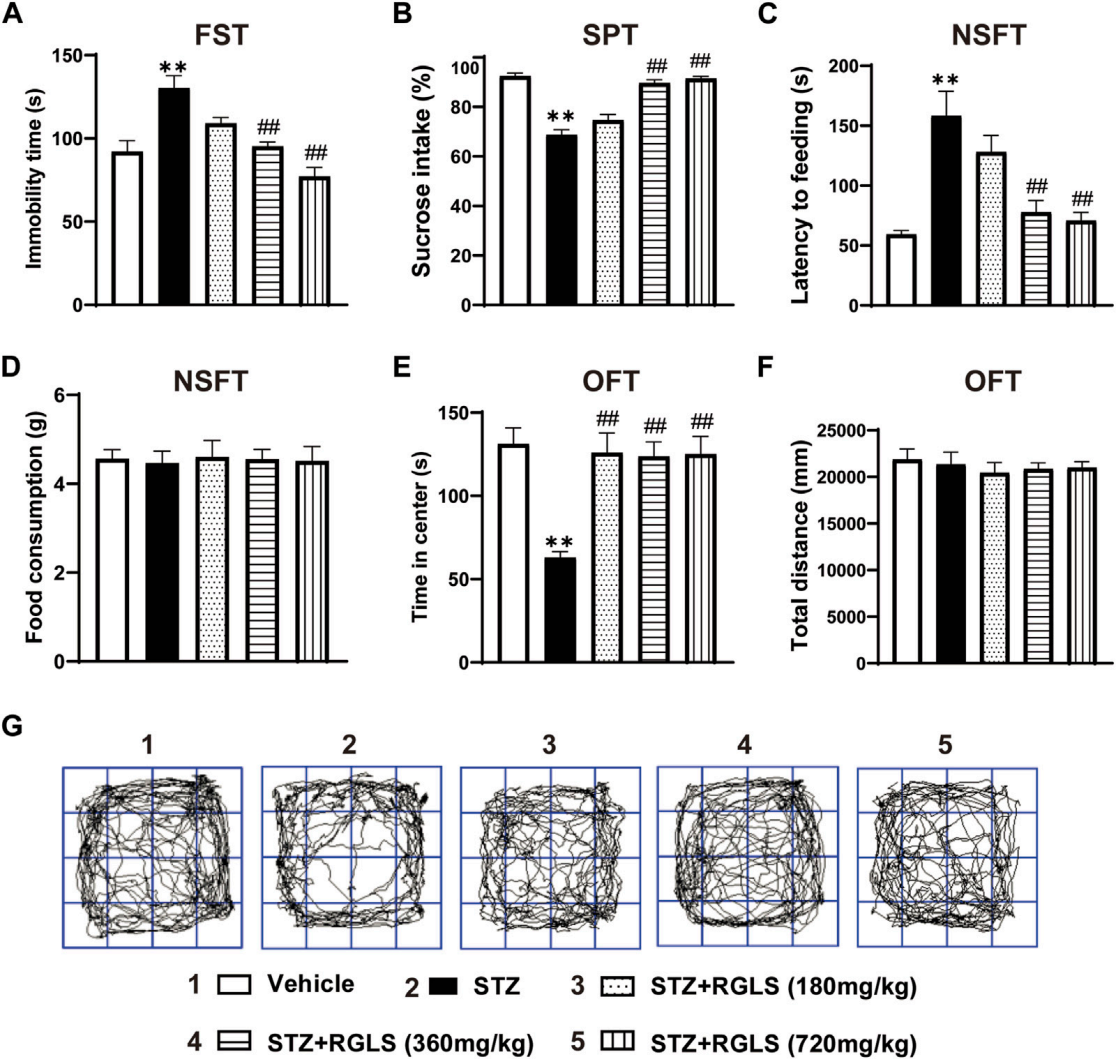
RGLS ameliorates ICV-STZ-induced depression- and anxiety-like behaviors. **(A)** The immobility time in the FST. **(B)** The sucrose intake in the SPT. **(C)** The latency time to feeding in the NSFT. **(D)** The food consumption in the NSFT. **(E)** The time in center in the OFT. **(F)** The total distance in the OFT. **(G)** Footprint pattern in the OFT. All data are presented as means ± SEM (n = 6). Compared to vehicle group, *p < 0.05, **p < 0.01; Compared to ICV-STZ group, #*p* < 0.05, ##*p* < 0.01.

ICV-STZ significantly prolonged immobility time in the FST (F_(4, 25)_ = 13.73, *p* < 0.001, Figure 2A), decreased sucrose intake in the SPT (F_(4, 25)_ = 44.16, *p* < 0.001, Figure 2B), and increased the latency time to feed in the NSFT (F_(4, 25)_ = 11.54, *p* < 0.001, Figure 2C), indicating depression-like behaviors in ICV-STZ rats. There was no significant difference in food consumption in the NSFT among all groups when excluding ingestion bias (F_(4, 25)_ = 0.0313, *p* > 0.99, Figure 2D). The administration of RGLS (360 and 720 mg/kg) prevented despair- and anhedonia-like behaviors that were induced by ICV-STZ (Figures 2A–D). For anxiety-like behavior, ICV-STZ markedly decreased the time spent in the center in the OFT, which was reversed by 360 and 720 mg/kg RGLS (F_(4, 25)_ = 9.41, *p* < 0.001, Figure 2E). There was no significant difference in the total distance travelled among groups, indicating that exposure to ICV-STZ and RGLS did not impair general motor ability in rats (F_(4, 25)_ = 0.2759, *p* = 0.8908, Figures 2F,G). These results indicated that 360 and 720 mg/kg RGLS alleviated depression- and anxiety-like behaviors that were induced by ICV-STZ.

### 3.2 RGLS inhibits inflammation and NF-κB/NLRP3 pathway activation in the mPFC in ICV-STZ rats

**FIGURE 3:**
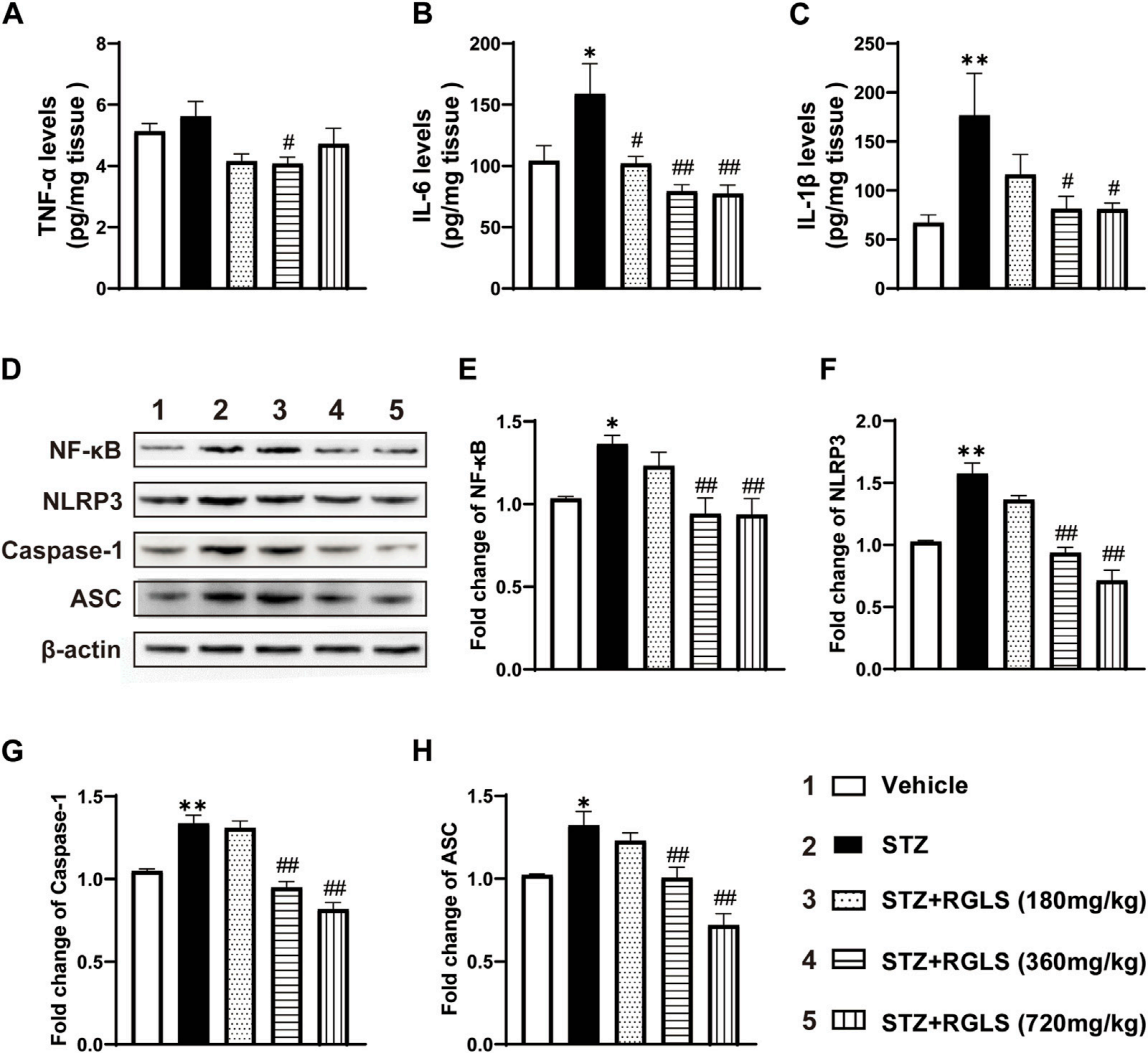
RGLS inhibits inflammation and NF-κB/NLRP3 pathway activation in the mPFC in ICV-STZ rats. **(A–C)** The levels of TNF-α, IL-6 and IL-1β in the mPFC (n = 5–6). **(D)** Representative western blots showing the expression of NF-κB, NLRP3, Caspase-1 and ASC in the mPFC. **(E–H)** Quantitative analysis of the proteins for NF-κB, NLRP3, Caspase-1 and ASC in the mPFC (n = 6). All data are presented as means ± SEM. Compared to vehicle group, *p < 0.05, **p < 0.01; Compared to ICV-STZ group, #*p* < 0.05, ##*p* < 0.01.

Depression-like behavior in rodents highly correlates with inflammatory responses in the central nervous system, and NF-κB/NLRP3 pathway activation is generally considered the initial inflammatory response. We examined proinflammatory factor and protein expression levels of several components of the NF-κB/NLRP3 pathway. Compared with rats in the vehicle group, TNF-α in the mPFC increased in ICV-STZ rats, but this increase was not statistically significant. RGLS treatment (360 mg/kg) reversed the ICV-STZ-induced increase in TNF-α expression (F_(4, 25)_ = 3.231, *p* < 0.05, Figure 3A). Similarly, ICV-STZ significantly increased IL-6 and IL-1β in the mPFC. The administration of RGLS (180, 360, and 720 mg/kg) reversed the ICV-STZ-induced increase in IL-6 (F_(4, 25)_ = 6.325, *p* < 0.05 or *p* < 0.01), and 360 and 720 mg/kg RGLS reversed the ICV-STZ-induced increase in IL-1β (F_(4, 24)_ = 4.362, *p* < 0.05, Figures 3B,C).

The NLRP3 inflammasome contains NLRP3 protein, adapter protein ASC, and caspase-1 (Shao BZ et al., 2015). NF-κB activates pattern recognition receptors and cytokine receptors license NLRP3 inflammasome activation by regulating NLRP3 expression (Bauernfeind et al., 2009). NF-κB, NLRP3, caspase-1, and ASC expression in the mPFC significantly increased in ICV-STZ rats compared with the vehicle group (*p* < 0.05 or *p* < 0.01, Figures 3D–H). These alterations were reversed by RGLS treatment (360 and 720 mg/kg) in ICV-STZ rats, and inflammation-related protein expression returned to levels of the vehicle group (NF-κB, F_(4, 25)_ = 6.286; NLRP3, F_(4, 25)_ = 34.22; caspase-1, F_(4, 25)_ = 35.67; ASC, F_(4, 25)_ = 15.43, *p* < 0.05 or *p* < 0.01, Figures 3D–H). These results indicate that RGLS exerts antiinflammatory effects by suppressing the NF-κB/NLRP3 pathway.

### 3.3 RGLS inhibits the activation of microglia in the mPFC in ICV-STZ rats

**FIGURE 4:**
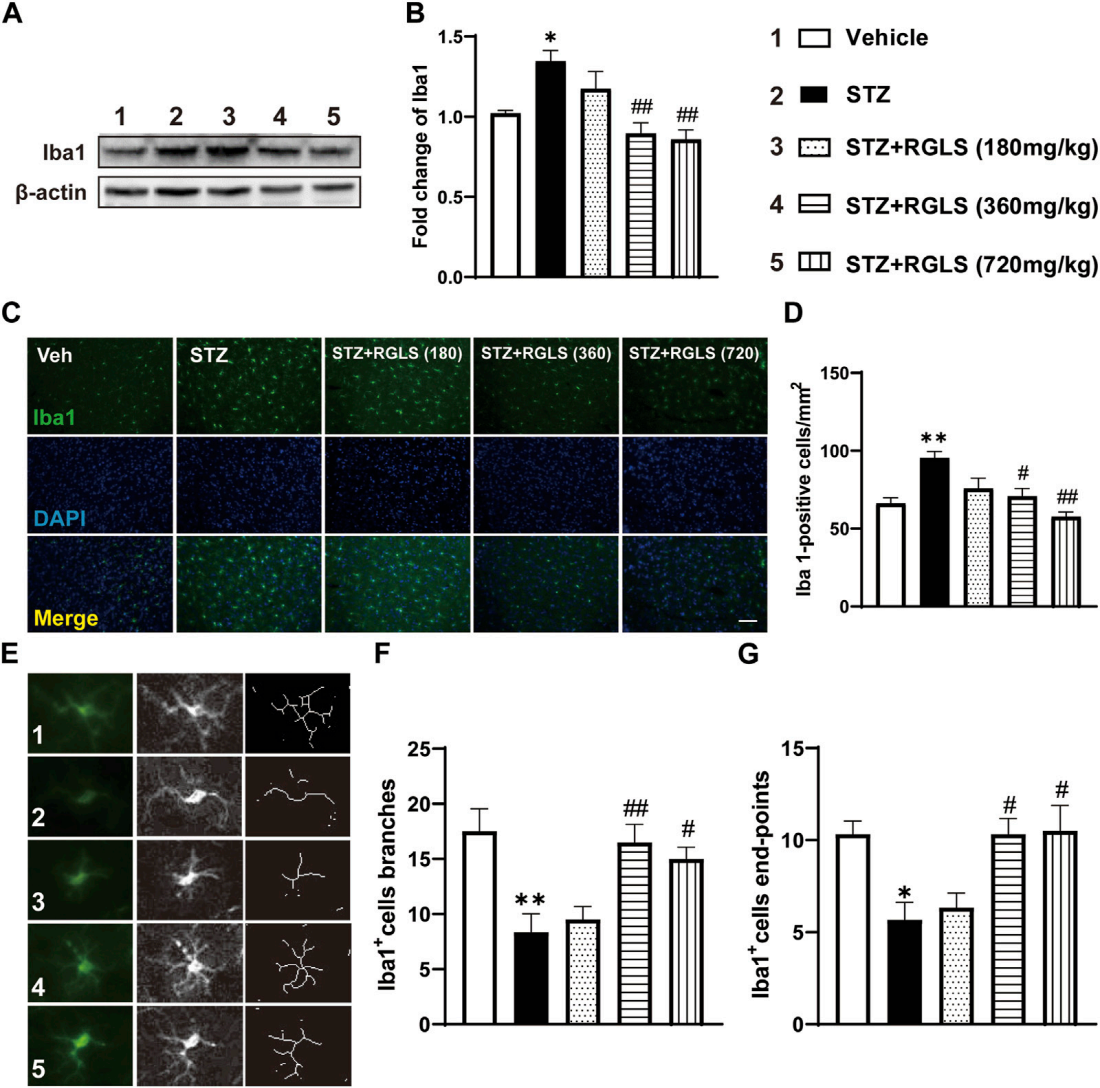
RGLS inhibits the activation of microglia in the mPFC in ICV-STZ rats. **(A)** Representative western blots showing the expression of Iba1 in the mPFC. **(B)** Quantitative analysis of the proteins for Iba1 (n = 6). **(C)** Representative immunofluorescence staining of Iba1 in the mPFC (scale bar = 100 µm). **(D)** Quantitative analysis of Iba1-positive cells number per mm^2^ (n = 6). **(E)** Representative image of microglia morphological alterations. **(F)** Quantitative analysis of branches number for each microglia. **(G)** Quantitative analysis of end-points for each microglia (n = 6). All data are presented as means ± SEM. Compared to vehicle group, *p < 0.05, **p < 0.01; Compared to ICV-STZ group, #*p* < 0.05, ##*p* < 0.01.

Microglia play an important role in neuroinflammation. We examined the activation of microglia in the mPFC to determine the potential antidepressant mechanism of action of RGLS. Functional and morphological features of microglia were evaluated by Western blot and the immunofluorescence staining of Iba1, a microglia-specific calcium-binding protein. Iba1 expression (F_(4, 25)_ = 8.558, *p* < 0.05, Figures 4A,B) and Iba1-positive cells (F_(4, 25)_ = 9.093, *p* < 0.01, Figures 4C,D) in the mPFC significantly increased in ICV-STZ rats, which were completely reversed by RGLS treatment (360 and 720 mg/kg). The morphology of microglia in the mPFC showed the significant attenuation of branch (F_(4, 25)_ = 7.122, *p* < 0.01) and end-point numbers (F_(4, 25)_ = 6.219, *p* < 0.01) in ICV-STZ rats, which were reversed by RGLS treatment (360 and 720 mg/kg, Figures 4E–G). These data indicated that RGLS treatment reversed ICV-STZ-induced pathological activation and morphological alterations of microglia in the mPFC.

### 3.4 RGLS exerts a protective effect against ICV-STZ-induced neuroplasticity damage in the mPFC

**FIGURE 5:**
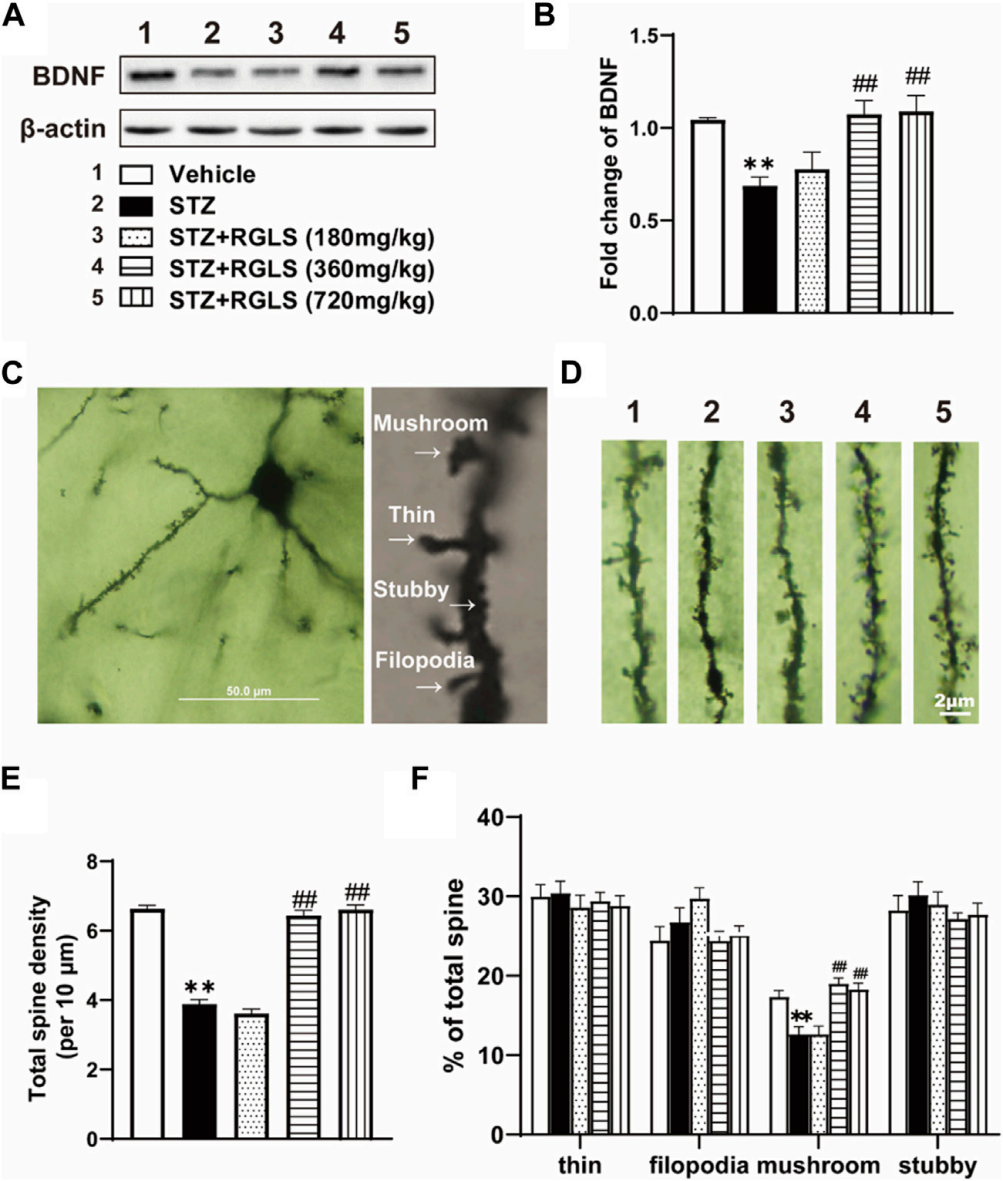
RGLS exerts a protective effect against ICV-STZ-induced neuroplasticity damage in the mPFC. **(A)** Representative western blots showing the expression of BDNF in the mPFC. **(B)** Quantitative analysis of the proteins for BDNF in the mPFC (n = 6). **(C)** Golgi staining of mPFC pyramidal neuron and four type of dendritic spine. **(D)** Representative image of dendritic spine in the mPFC (scale bar = 2 μm). **(E)** The number of total dendritic spine per 10 μm (n = 15 dendrites from 5 brains in each group). **(F)** The proportion for each type in the dendritic spine (n = 15 dendrites from 5 brains in each group). All data are presented as means ± SEM. Compared to vehicle group, *p < 0.05, **p < 0.01; Compared to ICV-STZ group, #*p* < 0.05, ##*p* < 0.01.

The central inflammatory response is associated with impairments in neuronal plasticity. BDNF levels were measured, and Golgi staining was performed to evaluate synaptic plasticity in the mPFC. BDNF expression in the mPFC significantly decreased in the ICV-STZ group, which was reversed by 360 and 720 mg/kg RGLS (F_(4, 25)_ = 7.545, *p* < 0.01, Figures 5A,B). Golgi staining of the mPFC was used to detect the damage of dendritic spines in ICV-STZ rats (Figures 5C,D). Neuronal dendrites with significant spine loss (F_(4, 70)_ = 150.9, *p* < 0.01, Figure 5E) and less of a mushroom type (F_(4, 70)_ = 14.93, *p* < 0.01, Figure 5F) were detected in the mPFC in the ICV-STZ group. RGLS (360 and 720 mg/kg) prevented these neuronal impairments in the mPFC in ICV-STZ rats. Altogether, these results suggest that ICV-STZ damages synaptic plasticity in the mPFC, which is significantly reversed by RGLS.

## 4 Discussion

The present study demonstrated that treatment with RGLS significantly ameliorated depression- and anxiety-like behaviors that were induced by ICV-STZ on day 7 in rats. Additionally, ICV-STZ significantly upregulated levels of inflammatory cytokines, NF-κB/NLRP3 inflammation-related signaling pathways, and microglia activation processes in the rat mPFC, and these increases were reversed by treatment with RGLS. We also found that RGLS significantly ameliorated ICV-STZ-induced reductions of BDNF levels and neuronal synaptic plasticity in the mPFC. These results suggest that RGLS might be useful for the treatment of depression- and anxiety-like behaviors in the ICV-STZ rat model of sAD. The mechanism of action of RGLS appears to involve the amelioration of inflammatory responses and attenuation of synaptic plasticity in the mPFC.

ICV-STZ is a common animal model that is used to simulate human sAD, which causes cognitive dysfunction and neuropsychiatric symptoms in rodents (Grieb, 2016). Although the pathological mechanisms that underlie neuropsychiatric symptoms of sAD are complex, neuroinflammation and impairments in neuroplasticity are important processes (Leng and Edison, 2021). The present study showed that ICV-STZ rats exhibited depression- and anxiety-like behaviors on day 7 (Figure 2). A significant neuroinflammatory response and synaptic loss and atrophy were also detected in the mPFC in ICV-STZ rats (Figures 3–5). These results indicate that ICV-STZ rats phenotypically and pathophysiologically resembled sAD, further implying the predictive value of this model to assess the efficacy of drug therapy.

Recently, researchers began investigating *G. lucidum* spores because they are a rich source of bioactive ingredients and to design methods to break the sporoderm (Soccol et al., 2016). Several studies showed that sporoderm-removed *G. lucidum* spore extract (RGLS) has higher activity than whole spores (Soccol et al., 2016; Li et al., 2020). The present study found that RGLS exerted significant antidepressant- and anxiolytic-like effects in ICV-STZ rats, reflected by the reversal of ICV-STZ-induced abnormal behavioral performance in the FST, SPT, NFST, and OFT. Our findings imply the potential treatment value of RGLS for the management of neuropsychiatric symptoms that are associated with AD (Figure 2). Previous studies showed that *G. lucidum* triterpenoids improved maternal separation-induced anxiety- and depression-like behaviors in mice by mitigating inflammation in the periphery and brain (Mi et al., 2022). Injections of *G. lucidum* polysaccharides led to a rapid and robust antidepressant-like effect in the chronic social defeat stress model of depression through a mechanism that involved the inhibition of proinflammatory cytokine levels and elevation of BDNF (Li H. et al., 2021). These results indicate that *G. lucidum* has anti-inflammatory and neuroprotective actions that might be associated with its antidepressant effects. Therefore, we explored the pharmacological mechanism of action of RGLS in ICV-STZ rats from the perspective of neuroinflammation and neuroplasticity.

Neuroinflammation is crucial for the development of AD, demonstrated by high levels of inflammatory markers and the identification of AD risk genes that are associated with innate immune function (Calsolaro and Edison, 2016). Previous studies reported significant increases in TNF-α and IL-6 levels in serum and brain tissue in AD patients (Fillit et al., 1991; Strauss et al., 1992). Cytokines, such as TNF-α, are able to upregulate the expression of key enzymes of neuronal Aβ formation through the activation of NF-κB signaling (Chen et al., 2012). NF-κB is considered a prototypical proinflammatory signaling pathway (Lawrence, 2009) that is necessary for NLRP3 inflammasome activation (Bauernfeind et al., 2009). The NLRP3 inflammasome is a multimeric protein complex that consists of caspase-1, ASC, and the danger sensor protein NLRP3. NLRP3 signaling then triggers the production of proinflammatory mediators, including IL-1β (Milner et al., 2021). The activation of NF-κB and the NLRP3 inflammasome is closely linked to AD via neuroinflammation (Halle et al., 2008). Interestingly, NLRP3 inflammasome-mediated neuroinflammation is also a key factor that is relevant to depression (Roy et al., 2023). Thus, targeting these proteins could be a rational strategy to treat neuropsychiatric symptoms that are associated with AD (Thawkar and Kaur, 2019). The present results showed that IL-1β, IL-6, and NF-κB/NLRP3 inflammatory signaling pathway proteins increased in the mPFC in ICV-STZ rats when they exhibited depression- and anxiety-like behaviors, suggesting that ICV-STZ-induced neuroinflammation might be a potential pathological mechanism by which these abnormalities occurred. Importantly, treatment with RGLS reversed ICV-STZ-induced neuroinflammation, reflected by the normalization of cytokine, NF-κB, NLRP3, caspase-1, and ASC expression in the mPFC, which might be an important mechanism that is related to RGLS-induced improvements in depression- and anxiety-like behaviors (Figure 3).

Microglia are primary immunocompetent cells that are found in the brain and express several Toll-like receptors (Ginhoux et al., 2010). The activation of Toll-like receptors subsequently results in the activation of NF-κB and production of proinflammatory cytokines (Kawai and Akira, 2007). Various immunohistological studies of human clinical samples found the presence of both traditional ramified resting microglia and amoeboid-shaped activated microglia in the AD brain (Davies et al., 2017). In early stages of AD, bipolar/rod-shaped microglia are evident in the affected brain region. As the disease progresses, amoeboid microglia predominate and are primarily localized in the hippocampus and cerebral cortex region where Aβ plaques and neurofibrillary tangles are commonly found (Plescher et al., 2018; Franco-Bocanegra et al., 2021; Wendimu and Hooks, 2022). Microglia also play an important role in the development and progression of depression by regulating neuroinflammation, synaptic plasticity, and the formation of neural networks (Jia et al., 2021). ICV-STZ was shown to induce mild reactive microgliosis in the dorsal hippocampus and PFC (Souza et al., 2022; Stanojevic et al., 2022). Consistent with previous studies, we found increases in the numbers and overactivation of microglia in the mPFC in ICV-STZ rats, and these abnormalities were reversed by RGLS (Figure 4).

Several studies have found impairments in synaptic plasticity in various neurodegenerative and neuropsychiatric diseases, including AD (Shao CY et al., 2011) and depression (Menard et al., 2016). Dendritic spine loss in the neocortex has been found in both AD patients and depression patients (Christoffel et al., 2011; Serrano-Pozo et al., 2011). BDNF, a neurotrophic growth factor, and its specific receptor TrkB play important roles in regulating spine density and spine shape (Yamada and Nabeshima, 2003; Tapia-Arancibia et al., 2008). The neurotrophic theory of depression stipulates that low levels of BDNF increase vulnerability to stress (Duman et al., 1997). In the mPFC in rats with ICV-STZ-induced depression-like behavior, we found a decrease in BDNF levels, accompanied by a reduction of mushroom-type dendritic spines. Mushroom spines are the main type of dendritic spines that perform physiological functions (Tackenberg et al., 2009). Treatment with RGLS protected the mPFC against synaptic plasticity deficits that were induced by ICV-STZ (Figure 5).

## 5 Conclusion

The present study investigated effects of RGLS on ICV-STZ-induced depression- and anxiety-like behaviors and the underlying mechanism. RGLS exhibited antidepressant and anxiolytic potential by suppressing cytokine levels and microglia overactivation and preserving synaptic plasticity in the mPFC. These results suggest that RGLS may be a potential therapeutic agent for the prevention and treatment of AD-related depression.

## Data Availability

The original contributions presented in the study are included in the article/Supplementary material, further inquiries can be directed to the corresponding authors.

## Abbreviations

AD, Alzheimer’s disease; *G. lucidum, G. lucidum*; RGLS, sporoderm-removed *G. lucidum* spore; ICV-STZ, intracerebroventricular injected with streptozotocin; sAD, sporadic Alzheimer’s disease; Aβ, beta-amyloid; mPFC, medial prefrontal cortex; NF-κB, nuclear factor kappa B; NLRP3, NOD-like receptor containing pyrin-domain 3; OFT, open field test; SPT, sucrose preference test; NSFT, novelty-suppressed feeding test; FST, forced swim test; ASC, apoptosis-associated speck-like protein; BDNF, brain-derived growth factor levels; Iba1, ionized calcium-binding adaptor molecule-1; TNF-α, tumor necrosis factor alpha; IL-6, interleukin-6; IL-1β, interleukin-1beta.
